# Hypertension knowledge, attitudes and perceptions among adults in the Navrongo Health and Demographic Surveillance Site: a mixed methods analysis

**DOI:** 10.1186/s12875-024-02469-3

**Published:** 2024-06-26

**Authors:** Ahaana Singh, Godfred Agongo, Samuel T. Chatio, Bernard Logonia, Cornelius Y. Debpuur, Patrick O. Ansah, Abraham R. Oduro, Kerstin Klipstein-Grobusch, Engelbert A. Nonterah

**Affiliations:** 1 Navrongo Health Research Centre, Ghana Health Service, Navrongo, Ghana; 2 International Health Academic Department, Global Health, Georgetown University, Washington, DC USA; 3 David Geffen School of Medicine, University of California, Los Angeles, CA USA; 4 Department of Biochemistry and Forensic Sciences, School of Chemical and Biochemical Sciences, C. K. Tedam University of Technology and Sciences, Navrongo, Ghana; 5 Department of Epidemiology, School of Public Health, CK Tedam University of Technology and Sciences, Navrongo, Ghana; 6 Research and Development Division, Ghana Health Service, Headquarters, Accra, Ghana; 7 Julius Center for Health Sciences and Primary Care, Julius Global Health, University Medical Center Utrecht, Utrecht University, Utrecht, the Netherlands; 8 Department of Epidemiology and Biostatistics, School of Public Health, Faculty of Health Sciences, University of the Witwatersrand, Johannesburg, South Africa

**Keywords:** Hypertension, Knowledge and Behavior, Perceptions, Health promotion, Ghana, H3Africa, AWI-Gen, Health and Demographic Surveillance

## Abstract

**Background:**

This study assessed knowledge, behaviors, and perceptions towards hypertension following community dissemination on cardiovascular disease (CVD) risk within the Navrongo Health and Demographic Surveillance Site in Northern Ghana.

**Methods:**

A cross-sectional mixed methods study was conducted among middle aged men and women following education on CVD and their risk factors. Knowledge and attitudes of participants regarding hypertension were measured in 310 participants using a survey tool and the resultant data was analyzed with descriptive statistics. Focus group discussions (FDG) were used to assess perceptions of 40 study participants on their lived experiences with hypertension. Recorded interviews were transcribed verbatim and coded into themes using Nvivo 12 software before thematic analysis.

**Results:**

Of the 310 surveyed participants, 54% were women and the mean age was 50 ± 6 years. The results showed that 84% of participants had heard about hypertension, 70% knew it was an increase in blood pressure and could be caused by excess salt intake, poor diet and physical inactivity. About 22.3% of participants were aware of the had hypertension. In terms of management, majority of the participants were aware that hypertension could be treated with antihypertensive medication and that untreated or uncontrolled hypertension could result in adverse health consequences. Few participants had ever had their blood pressure measured and did not access screening or healthcare care services and rather perceived the health system as inadequate to screen, and manage hypertension.

**Conclusion:**

Though, knowledge on hypertension was high, awareness of hypertension status and access to screening and healthcare services was low. Community beliefs and perceptions strongly influence treatment, and control of hypertension. Effective sustained community dissemination efforts addressing misperceptions could improve hypertension treatment and control.

**Supplementary Information:**

The online version contains supplementary material available at 10.1186/s12875-024-02469-3.

## Introduction

Hypertension is a leading modifiable risk factor for cardiovascular disease (CVD) across the globe affecting 1.3billion people in 2022 and 32.8% of deaths globally [1, 2]. About 80% of these occur in low- and middle-income countries, like Ghana [3]. In Ghana hypertension has experienced an increase of 28.6% between 2009 and 20,019 with an associated increase in CVD-related morbidity and mortality by 25% and 38% respectively within the same period [4]. While the increased incidence of hypertension has traditionally been seen as an urban concern, the rural–urban gap is closing, especially among older adults [5]. Controlling hypertension can mitigate CVD morbidity and mortality across Ghanaian communities. However, awareness and control of hypertension amongst Ghanaians between the ages above 18 years is observed to be low [6, 7].

The coverage area of the Navrongo Health and Demographic Surveillance Site (HDSS) has experienced a rapid increase in the prevalence of hypertension from 19.3% in 2009 [8] to 24.5% in 2015 [7]. The rising burden of hypertension in the area is associated with a low awareness and even lower levels of treatment and control of hypertension. Only 1 out of 10 hypertensive adults had achieved control in 2015 with more women than men likely to achieve control [7, 9]. While low literacy has been implicated in the low awareness levels, inadequate understanding of healthy lifestyle factors and insufficient health system strengthening in risk factor identification, surveillance and lack of access to counseling are also important identified factors [8]. The African-Wits-INDEPTH genomic study (AWI-Gen) was conducted between 2015 and 2016 to investigate the genomic and environmental risk to cardiometabolic diseases [10]. Following the successful implementation of the study in 2017, community feedback sessions were organized to improve awareness on CVD and associated risk factors at the community level and individually with the study participants [11]. The potential impact of this community feedback on participants’ knowledge, behaviors and perceptions about hypertension is yet to be assessed.

Although, the community feedback sessions unveiled an apparent lack of knowledge and awareness about hypertension amongst adults (40—60 years), objective measures of knowledge and awareness regarding hypertension have not been previously examined. By assessing existing knowledge, behaviors, and perceptions surrounding hypertension in this specific community, evidence-driven, contextual interventions can be developed and explored in order to overcome gaps in knowledge and promote healthier behaviors in an effort to reduce hypertension morbidity and mortality.

Accordingly, this study sought to generate information to identify targeted educational and health promotion interventions that can be developed and implemented. Specifically, this mixed-methods paper focuses on determining the levels of hypertension knowledge amongst adults aged 40 to 60 years in the Navrongo HDSS coverage area in Northern Ghana. Further, this study explored the attitudes, behaviors, and perceptions that may influence consequent hypertension management and prevention in the primary healthcare system in Northern Ghana.

## Methods

### Study design and setting

This study was an exploratory mixed-methods design consisting of a quantitative assessment of knowledge, attitudes and behaviors as well as a qualitative assessment of perceptions about hypertension in the Navrongo Health and Demographic Surveillance Site Ghana in 2021. The Study participants were a subset of participants recruited from the AWI-Gen study that was conducted from 2015 to 2020 under the auspices of the Human Heredity and Health in Africa (H3Africa) consortium [10]. The participants included in the AWI-Gen study were middle-aged women and men residing in the Navrongo HDSS coverage area and who were free of established CVDs (such as stroke, myocardial infarction, heart failure and peripheral vascular disease) and were not pregnant at the time of data collection. Other participants excluded were first-degree relatives of existing participants, and individuals with physical impairments that interfere with blood pressure measurement. This study, which took place between October and December, 2020, recruited a subset of AWI-Gen study participants who have received community feedback education on risk factors of cardiometabolic diseases (CMD) [11]. Participation in one arm (either qualitative or quantitative) of the study automatically excluded one from participation in the alternative arm.

### Sample size

The available participant pool from the AWI-Gen study formed the sample frame (*n* = 2016) from which the study participants were randomly selected to partake in either the quantitative arm or the qualitative arm of this study. To determine knowledge, attitudes, and behaviors through a quantitative study, 353 subjects (*n* = 353) were randomly selected to take part in the survey. This sample size was calculated based on hypertension prevalence of 24%, leading to a target sample size of *n*_*t*_ = 321. The formular used for the calculation is as follows:

$$n={Z}^{2} P(1-P)/{d}^{2}$$

where *n* = sample size, Z is the statistic corresponding to level of confidence, P is expected prevalence (This was 24% based on the prevalence gotten from the AWI-Gen study).

In order to account for potential non-response, the target sample size (*n*_*t*_ = 321) was increased by 10% (*n* = 354). The randomized sample was divided equally amongst the four geographical zones of the Kasena-Nankana East Municipal and Kasena-Nankana West District; 353 participants were surveyed from the North, South, and East and West zones of the Navrongo HDSS coverage area. A total of 310 out of the 353-sample size complete responses that were used for the quantitative analyses.

For the qualitative study, 40 individuals were randomly selected from within the AWI-Gen study participants for the focus group discussions (FGDs). These participants were divided into four groups with 10 individuals from each of the four zones of the Navrongo HDSS coverage area in order to ensure geographical representation and views. Four FGDs were conducted with women and men to explore their perceptions about hypertension. Men and women were brought together for the discussions since they shared a common interest – hypertension, which was not agender sensitive topic and in an effort to stimulate broad-based engagement and expose divergent thinking amongst the participants. In doing so, participants thought about varied perspectives (across gender, occupation, and lifestyle) and provided more nuanced thoughts based on the ideas of others.

### Data collection procedures and statistical analysis

Considering the mixed-methodology approach of this research, data collection occurred in two separate arms.

#### Quantitative arm

Using a close-ended questionnaire, data measures on knowledge, attitudes, and behaviors (practices) of hypertension and associated risk factors were collected (Supplementary material). The sampled individuals were approached, informed consent obtained before the survey was administered. The questionnaire was administered verbally in the person’s preferred language by a fieldworker using an encrypted tablet equipped with KoBoToolbox data collector (computer assisted participant information). In the case electronic documentation of responses was not possible, each fieldworker was equipped with printed questionnaires to administer and subsequently record into the electronic survey when access was restored. Data from KoBoToolbox software were exported into STATA version 14 (StataCorp LP, Texas, USA), for data management and analyses. Categorical data were summarized using frequencies and corresponding percentages while normally distributed continuous data were summarized using means and standard deviations.

#### Qualitative arm

Two trained research assistants with previous experience in qualitative interviewing techniques conducted the interviews using a topic discussion guide. Appointments were booked with participants for a suitable date, time and venue for the FGDs. The discussions were done in the two main local languages in the area (Kasem and Nankani) and within the community to ensure open comfortable discussions. One research assistant moderated the discussions, while the other one recorded the discussions and also took notes. Following group-wide consent, the FGD was recorded on an encrypted audio-recording device. The recording was subsequently transcribed verbatim into English. Since interviews were conducted in the local languages and transcribed verbatim, some editing had to be done to improve the flow and understanding of the text. Thus, the transcripts were edited by the lead investigator. However, this was done without changing the original meaning of statements made by participants. The transcripts were edited merely to correct grammatical mistakes to make them readable before data coding and to also ensure high quality of data.

Qualitative data analyses were rooted in phenomenology, exploring how individuals perceive and give meaning to hypertension. A codebook was developed using a combination of established categories based on the original research questions and themes that emerged from the data. The transcripts were imported and coded using NVivo software version 12 before thematic analysis. To ensure a fair interpretation of the data, the transcripts were initially coded independently by two researchers. Guided by the objectives of the study, the coding process involved a critical review of each transcript to identify emerging themes from the data. The two coders then met to compare their independently-identified themes. They resolved any divergence by re-reading the relevant sections of the transcripts together, and agreed on the best fit interpretation of the data. The themes discussed in the results section are supported by relevant quotes from the transcripts.

## Results

### Quantitative findings

#### Description of the study participants

We analyzed data for 310 participants with their sociodemographic characteristics and self-awareness of hypertension summarized in Table 1. About 54% of the survey participants were women and the average age of the entire population was 50 ± 6 years (range, 45—65 years). Sixty-nine participants representing 22.3% were aware of their hypertensive status while three (0.97%) of those participants reported having at least one or more co-existing cardiometabolic disease, such as diabetes.

**Table 1:** Sociodemographic characteristics of the study participants

Variables	Frequency (*n*_f_ = 310)	Percentage (%)
Sex		
* Male*	143	46.1
* Female*	167	53.9
Age		
* 40–44 years*	52	16.8
* 45–49 years*	84	27.1
* 50–54 years*	63	20.3
* 55–60 years*	111	35.8
Geographic zone		
* North*	81	26.1
* South*	75	24.2
* East*	81	26.1
* West*	73	23.6
Education Level		
* None*	232	74.8
* Primary*	50	16.1
* Secondary*	24	7.74
* Tertiary*	4	1.29
Employment		
* Unskilled*	111	35.8
* Skilled*	199	64.2
Household size		
* 1–5*	116	37.4
* 6–10*	172	55.5
* 11* +	22	7.1
Has elevated Blood Pressure		
* Yes*	69	22.3
* No*	241	77.7
Has other CMB condition (s)		
* No*	302	97.4
* Yes*	3	0.97
*Unknown*	5	1.61

*CMB* Cardiometabolic

### Awareness, knowledge, attitudes and behaviors

About 84% of the respondents had heard of hypertension before and 69% knew that hypertension was elevated blood pressure. In general, most respondents could identify one or more of potentially associated symptoms and half of the respondents (56%) did not know that HTN could be asymptomatic (Table 2). Among those who were familiar with hypertension, 97% believed that it could be controlled with treatment. Within this subset, the vast majority (95%) of respondents believed that orthodox medications were the best type of treatment. Nearly all respondents believed that there are short- and long-term adverse health consequences of hypertension.

**Table 2:** Proportions of self-reported awareness of hypertension status, basic knowledge and beliefs about hypertension amongst participants

Knowledge of hypertension	Frequency (*n* = 310)	Percentage (%)
Have you ever heard of hypertension?		
* Yes*	260	83.87
* No*	50	16.13
What is the definition of hypertension?		
* High blood pressure*	191	68.95
* Excessive stress or worry*	71	25.63
* Lack of attention*	5	1.81
* Don’t know*	10	3.61
What causes hypertension?		
* Food poisoning*	186	67.15
* Bad or evil spirits*	167	60.29
Is hypertension hereditary?		
* Yes*	102	36.82
* No*	93	33.57
* Don’t know*	82	29.60
What are the symptoms of hypertension?		
* Headache*	114	41.16
* Palpitations*	59	21.30
* Fever*	3	1.08
* Chest pain*	48	17.33
* Faintness*	3	1.08
* Sudden collapse*	13	4.69
* Sudden stroke*	4	1.44
* Other*	1	0.36
* Don’t know*	32	11.55
Can one have hypertension without any symptoms?		
* Yes*	122	44.04
* No*	155	55.96
**Knowledge on treatment**		
Can hypertension be controlled with treatment?		
* Yes*	269	97.11
* No*	8	2.89
Type of treatment to be used (*n* = 269)		
* Traditional medicines*	8	2.97
* Orthodox medications*	255	94.80
* Herbal remedies*	5	1.86
* Other*	1	0.37
Consequences of untreated hypertension		
* None*	1	0.36
* Stroke*	65	23.47
* Heart failure*	30	10.83
* Death*	170	61.37
* Other*	1	0.36
* Don’t know*	10	3.61

### Health seeking behaviors and knowledge of lifestyle risk factors

Presented in Table 3 is information on health seeking behaviors and lifestyle risk factors of all respondents (including those who did not report being hypertensive as well as those unfamiliar with hypertension). The vast majority of respondents (93%) indicated that they are concerned with their overall health. However, only 43% reported that they attend clinics regularly. In general, most respondents (91%), visit the clinics only for injuries or instances of feeling generally unwell. About 45% respondents recall having had their blood pressures measured by health personnel in the health facility between 2 to 6 times annually. Respondents identified high levels of salt (98%), oil (89%), and rice (88%) consumption as well as physical inactivity (71.3%) as common lifestyle risk factors that could cause hypertension.

**Table 3:** Healthcare Seeking behaviors and knowledge of lifestyle risk factors

Behavior Category	Frequency ( n = 310)	Percentage (%)
Are you concerned with your overall health?		
* Yes*	289	93.23
* No*	19	6.13
* Somewhat*	2	0.65
Do you attend clinics regularly?		
* Yes*	132	42.58
* No*	178	57.42
Why do you typically visit the clinic?		
* Routine visit*	28	9.03
* Sick/injury visit*	282	90.97
Do you use alternative (i.e. traditional) medicines?		
* Yes*	38	12.26
* No*	272	87.74
Have you had your blood pressure measured?		
* Yes*	141	45.48
* No*	169	54.52
How often do you have your blood pressure measured? (*n* = 141)		
* 2–4 times a month*	6	4.26
* Once a month*	18	12.77
* 6–11 times a year*	2	1.42
* 2–6 times a year*	63	44.68
* Once a year*	51	36.17
* Other*	1	0.71
***Lifestyle risk factors***		
Do you take salt with your food?		
* Always*	266	85.81
* Sometimes*	37	11.94
* Never*	7	2.26
Do you cook your food with oil?		
* Always*	103	33.23
* Sometimes*	170	54.84
* Never*	37	11.94
How often do you eat fruits and vegetables?		
* 1–4 times a day*	5	1.61
* 1–5 times a week*	304	98.06
* Never*	1	0.32
How often do you eat meat?		
* Every day*	2	0.65
* 2–5 times a week*	43	13.87
* Once a week*	198	63.87
* Never*	67	21.61
How often do you eat rice?		
* Every day*	18	5.81
* 2–5 times a week*	255	82.26
* Once a week*	36	11.61
* Never*	1	0.32
Do you consume alcohol?		
* Yes*	149	48.06
* No*	161	51.94
Do you smoke?		
* Yes*	54	17.42
* No*	256	82.58
Do you exercise regularly?		
* Yes*	89	28.71
* No*	221	71.29

Regular exercise is defined as a minimum of 150 min of moderate-to-vigorous exercise per week

### Qualitative findings

Further exploration regarding knowledge/awareness, understanding and perception of hypertension was done in the qualitative interviews as discussed below. A total of 40 individuals made up of 19 women and 21 men were involved in the qualitative arm of the study. The main themes were awareness and understanding of hypertension, perceived causes of hypertension, Perceived strategies to prevent hypertension while the sub-themes discussed were resources to take care of blood pressure and capability of managing blood pressure.

### Awareness and understanding of hypertension

In the qualitative interviews, participants viewed hypertension as a very bad disease that could kill people within a short time if care was not taken. They described hypertension as a sudden heart attack, which usually happens when a person experiences an unusual heartbeat, thus leading to high blood pressure.

*R4: It (referring to hypertension) can kill you within a short period of time, it can throw you down and you will be acting like someone who has epilepsy. Hypertension is not a good disease.* (FGD - males and females, Paga)

*R5: For our local languages we call it (referring to hypertension) heart attack. When it attacks you, you cannot breathe well and they quickly send you to health center for treatment. If not, you will die within a short period of time.* (FGD - males and females, Kandiga)

*R1: That disease kills very fast, it can throw you down suddenly, that disease is very bad, hypertension kills very fast.* (FGD - males and females, Paga)

### Perceived causes of hypertension

Discussants mentioned various causes of hypertension, which included consumption of certain foods in the study area. Participants perceived that eating unhealthy foods such as sweets, oil, fats, spices, and too much “Maggi” (a spice mixture) could all cause hypertension. Participants believed that most people in their communities were suffering from blood pressure or hypertension because they were eating these “unhealthy” foods. Some individuals also held that eating pig and donkey meat as well as excessive alcohol intake could cause hypertension.

*R5: I think that the pig and donkey meat were not there and hypertension was not also there but now those meat are there and they are the cause of hypertension.* (FGD - males and females, Mirigu)

*R7: Drinking too much alcohol can also cause it (referring to hypertension).* (FGD - males and females, Paga)

*R8: Our section of Kandiga community there are a lot of people that are suffering from blood pressure and the reason is that they eat a lot of unhealthy foods like oil, fats and also drinking of alcohol can cause the disease*. (FGD - males and females, Kandiga)

*R8: I agree with what he said because the sweets are plenty, in the past they were using only manure on the farm lands, but now vegetables like Kenaf, Roselle and many others, it is fertilizer they add to it. These can all cause hypertension.* (FGD - men and women, Paga)

Some participants reported that consumption of foreign foods imported into Africa also caused hypertension for many people. As these participants expressed it:

*R3: What I also have to say is that everything comes from the foreigners or white people because we the Africans, we cannot have our own foods. You see some foods that we prepare comes from the white people so I think they should always look at it very well whether it will increase our health conditions or not before they allow people to eat it.* (FGD - males and females, Kandiga)

*R2: I think the foods we eat now, which comes from the foreign land has brought about hypertension in recent times.* (FGD - males and females, Mirigu)

Other participants were of the view that chemicals used for growing foods could also cause hypertension as described in the excerpts below:

*R5: I think that the kinds of foods that are coming recently bring about hypertension. The reason is that at first when you farm tomatoes, you will be watering the tomatoes till it is time for harvesting but now they normally use medicine to spread them to be ready to harvest in order to get high prices. I think that it is a cause of hypertension because we eat chemicals.* (FGD - males and females, Mirigu)

*R7: I think the leafy vegetables are good but the chemicals, that they apply for them to grow fast so that they can harvest is the main problem.* (FGD - males and females, Kandiga)

Some participants also believed that situational factors that can cause emotional stress, such as loss of a loved one or poverty, could lead to hypertension. According to these individuals, when such unfortunate events happened, it could get the affected persons to think so much, which could cause hypertension. The following extracts exemplified how these participants shared their views on the issue:

*R2: I think hypertension is the process where you are thinking beyond normal due to a problem. When it happens like that, your mind is not at one place and that will make such a person to have hypertension.* (FGD - males and females, Mirigu)

*R6: At first, we were not dying as the way we are dying now. You will be sitting and they will inform you that this your brother has passed away. Your heart will beat and your heart beating will also cause another problem, which is the hypertension.* (FGD - males and females, Kandiga)

### Perceived strategies to prevent hypertension

Various strategies were recommended by participants to prevent hypertension. For instance, participants believed that consumption of traditional foods and vegetables, such as Kenaf, Amaranthus, and bean leaves could reduce the occurrence of hypertension in the study area. Reduced alcohol intake and avoiding foods that contained too many spices were also recommended by participants to help prevent people from getting hypertension.

*R2: If you want to control blood pressure unless we stop habits that are not good. We should not drink alcoholic, avoid oil foods and those are the things that can prevent us from getting blood pressure or control blood pressure*. (FGD - males and females, Kandiga)

*R7: Our olden food such as leaf vegetables such Kenaf, Amaranthus, bean leaves etc. and millet can prevent hypertension because those food are healthier for our system.* (FGD - males and females, Mirigu)

*R5: What we can do to prevent it (referring to hypertension), is that if you were taking in more alcohol, you have to limit how you drink alcohol, if you were eating fresh meat; you have to limit how you take in fresh meat. If you were taking in more Maggi you have to limit how you consume Maggi.* (FGD - males and females, Paga)

A participant held that the use of moringa plant to prepare food was one strategy that could control hypertension.

*R7: To be honest, with the moringa, if you agree to eat it, it is very good. It is only the moringa that can prevent hypertension. If you use it (referring to moringa) to prepare foods, I think it will control BP.* (FGD - males and females, Paga)

The minimization of screaming and shouting were also perceived as potential measures that could prevent people from getting hypertension. As one participant expressed it:

*R2: What we can do is, we should stop screaming at children and shouting at people. Shouting all the time can cause one to have a problem in the heart. So, we should talk in the low tone and that will help.* (FGD - males and females, Kanania)

Frequent health check-ups were recommended as an important way to help minimize the occurrence of hypertension according to some participants.

*R5: It is important we have the patience to go and check all the time. As for me, I go to Saga health facility to check my BP.* (FGD - males and females, Paga)

*R2: To be honest, if we are able to frequently check ourselves, it is a good thing to help prevent the disease.* (FGD - males and females, Kanania)

### Resources to take care of blood pressure

Views of participants were also solicited regarding whether they had the necessary resources to help manage hypertension at home. Discussants mentioned various local herbs and resources, such as neem leaves, dawadawa, and bitter leaves that could be used to manage the condition. The following quotes represent the views expressed by these participants on the issue:

*R5: When we wake up, we use neem leaves to cover yourself to see if you will get better, so that is what I know.* (FGD - males and females, Kanania)

*R3: If you notice you are not feeling well, you should boil neem leaves, after boiling, you should inhale the steam from the neem leaves; you should cover your head while you bind your head towards the pot to inhale the steam. After that, you can drink the neem water and also use it to prepare vegetable soup and eat.* (FGD - males and females, Kanania)

*R7: We should be using dawadawa as ingredients alone for cooking because I think that will help us a lot. We can also use the local herbs to treat our sickness.* (FGD - males and females, Kandiga)

### Capability of managing blood pressure

Participants noted that they did not have the capacity to manage hypertension or control their blood pressure because they did not have any medical devices resources at home or readily accessible for them to check their blood pressure (BP) regularly. They held that it was only at the health facility they could get the opportunity of checking to know their BP status.

*R8: That is what I already said that we don’t know what to do to control blood pressure neither the resources you are talking about.* (FGD - males and females, Kandiga)

*R7: We don’t have anything. I go to the hospital and they put something under my armpit to see what is wrong with me. Unless I go to the hospital for them to check and see what is wrong with me. Aside that we don’t have anything at home.* (FGD - males and females, Paga)

*R1: I don’t have a BP checking device to use at home.* (FGD - males and females, Kanania)

**Fig. 1:**
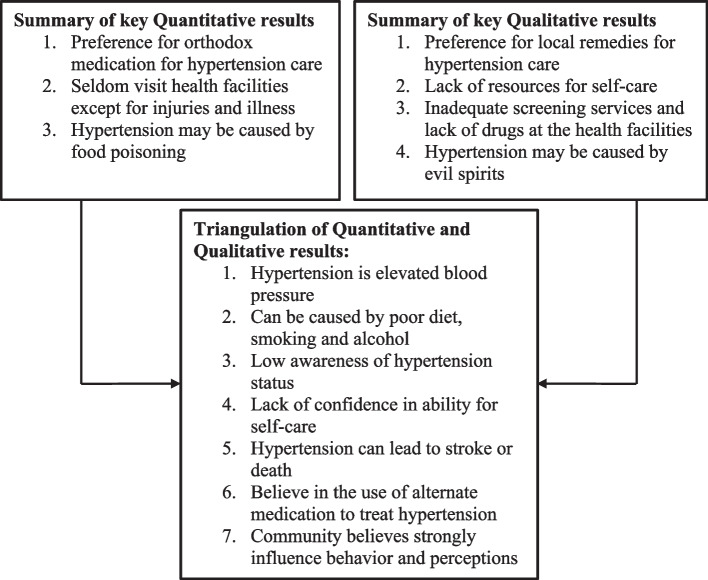
Triangulation of qualitative and quantitative results

The results of the qualitative analyses are summarized in Table 4 while triangulation of quantitative and qualitative results are indicated in Fig. 1.

**Table 4:** Summary of Qualitative Findings from FGDs

Knowledge on Hypertension	Causes of hypertension	Prevention and management of hypertension	Resources & capacity to manage hypertension at home
It is high blood pressure	Unhealthy foods (e.g., sweets, spices, oils, fats)	Consuming traditional foods and plants (e.g., green leafy vegetables, moringa, Kenaf)	Utilization of local herbs and remedies
It is very bad, potentially fatal disease	Too much meats (e.g., donkey, pig)	Reduced alcohol intake	Lack of necessary resources such as medication
Sudden heart attack characterized by an unusual heartbeat	Emotional stress	Reduced spice and oil intake	They are only able to do check up in the health facilities
	Excessive alcohol intake	Management of anger/stress	They observed that nurses do not have equipment for screening
	Exposure to agricultural chemicals	Frequent health check-ups	
	Foreign food (energy dense meals)		
	Excess salt in take

The overlapping message between the quantitative and findings are captured in Fig. 1. These include low awareness of hypertesnion status, high knowledge of common risk factors, the belive hypertension can lead to stroke or death and finally the believe that, there are alternative methods of managing hypertension.

## Discussion

This study set out to determine knowledge, attitude, and perceptions towards hypertension amongst middle-aged adults drawn from the AWI-Gen study in the Navrongo HDSS. The study determined that there were high levels of general knowledge about hypertension and it causes but low awareness of hypertension status across both arms of the study. Yet, both arms also indicated some awareness of relevant risk factors, such as an unhealthy diet, alcohol consumption, and increased emotional stress. In addition, the majority of participants reported having general concerns for their overall health; however, they lack confidence in their personal ability to control their blood pressure. Notably, the quantitative responses indicated a preference for orthodox medications, though acknowledge local remedies which was corroborated by the focus group discussions where alternative means for hypertension treatment such as use of herbal remedies and traditional foods were mentioned.

The prevalence of hypertension recorded by the AWI-Gen study in 2015 was 24.5% [7], which is higher than the 2009 recording of 19.3% [8]. Prior to the community sensitization and feedback, the AWI-Gen study had observed low levels of awareness, treatment and control of hypertension [7] and diabetes [12]. The level of awareness observed in this study was 22.3%, which is lower than that was reported before the sensitization. Following the community feedback sessions embarked by the AWI-Gen team [11], we were expecting to record a higher awareness. The low levels of hypertension awareness may be due to low literacy and inadequate understanding of lifestyle factors. Other reasons may include inadequate health system strengthening leading to low hypertension-related risk factor surveillance and hence further causes low awareness levels.

Consistent with our findings [13], in their study among lay hypertensives in a similar northern setting reported a perception that hypertension could be cured with herbal medication [13]. This underscores the need for health education to improve upon knowledge and understanding of hypertension and its associated management and complications. Studies on the effectiveness of health education interventions aimed to increase knowledge and consequent management and prevention of hypertension show immense promise globally. Various experimental and quasi-experimental studies that employed therapeutic patient education interventions have indicated improvements in lifestyle factors associated with hypertension [14, 15, 16, 17]. It’s important to note, however, that interventions across existing literature vary considerably in mode and scope of education employed. In general, educational interventions appear to most effectively improve blood pressure levels when supplemented with self-monitored blood pressure [15]. Self-Management of Blood Pressure, however, requires reliable access to at-home devices for measurement of blood pressure, which is often difficult to maintain in rural settings like Kasena Nankana Districts.

Our recent findings highlight the difficulty in educational programs achieving progress without augmenting it with other integrated programming. Blood pressure control also appears to be correlated with the presence of other risk factors. A study conducted in south-east Nigeria that employed educational interventions without supplementation with self-monitored blood pressure saw significant improvements in key lifestyle and risk factors associated with hypertension; specifically, physical activity, sleep pattern and quality, substance use, healthy diet, and medication adherence [16]. We acknowledge that having returned to these participants 5 years after the initial education prior to this study without supportive resources may also affect their ability to self-manage blood pressure. The time lag could also affect their knowledge on some of the topical areas discussed in this paper.

Moreover, several of these lifestyle factors have proven to be predictors of BP control within urban Ghanaian communities. In particular, increased physical activity, alcohol and smoking abstinence, increased consumption of fruits and vegetables, and reduced consumption of carbohydrates, meat, and fat, appear to have a positive influence on blood pressure control [18]. In addition, verbal educational interventions have shown improvements in health literacy and consequent adherence to medication among individuals with hypertension [19]. We recommend that further research is conducted to fully understand the knowledge attitudes, behaviours and perceptions of community members on hypertension and to fully explore the role of health systems as well as community facilitators and barriers to improved screening and management of hypertension.

### Strengths and limitations

In conjunction with the aforementioned quantitative findings, the complementary qualitative data present opportunities for educational interventions to shape targeted information for the specific needs of the studied communities. This was an appropriate means of data collection because it allowed community members to collectively reflect on their feelings towards hypertension. This methodology provided the research team with insights on community-wide ideologies, stigmas, and concerns regarding hypertension.

The qualitative approach also allowed the research team to witness how individuals discuss hypertension amongst one another, albeit in an unnatural environment, which has helped the team to have in-depth and normative information on the topic. Overall, the study gives us first-hand information on the impact of community feedback of results on participants. A key limitation is the risk of recall bias but this was mitigated as we verified reported hypertension status with previous and current blood pressure measurements. Social desirable responses can not be ruled out hence possibility of generating biased views. We also acknowledge that mixing men and women in the FGDs may likely to restrict women from sharing their views since study setting is patrilinear society. However, since the participants shared a common interest—hypertension, which is not gender-sensitive, we believe mixing them had a less likely impact the views they expressed.

## Conclusions

Based on the interpretation of our data, study participants appeared to be less aware of their hypertension status despite having some basic knowledge on hypertension, its associated causes and treatment options. Awareness and knowledge on hypertension is strongly influenced by community lived beliefs and perception. While community dissemination through education is a potential way of improving hypertension screening, medication and adherence, we advise that this should be done as a sustained process through the existing primary healthcare setup and not as a once-off research activity. Further research should focus on appropriate and sustainable methods of integrating health promotion and access to screening and management of hypertension in the community level through primary healthcare.

### Supplementary Information

Supplementary Material 1.

## Data Availability

Data used for this manuscript are available upon reasonable request from the corresponding authors.
