# Supplementary material for: Hypertension knowledge, attitudes and perceptions among adults in the Navrongo Health and Demographic Surveillance Site: a mixed methods analysis
Source: BMC Prim Care. 2024 Jun 26;25:229. doi: 10.1186/s12875-024-02469-3 (PMC11201899; doi:10.1186/s12875-024-02469-3)
Supplement: Supplementary file 1 — Supplementary Material 1. [file 12875_2024_2469_MOESM1_ESM.pdf]

Supplementary data

Appendix 1: Knowledge Attitude Perceptions Survey

Section 1: Knowledge on Hypertension

NO.	QUESTIONS AND FILTERS	CODING CATEGORIES	SKIP?
1	Have you ever heard about hypertension before?	YES ………………….……… 1<br>NO ………………………..… 2	→ 26
2	Where did you hear about hypertension for the first time?	THIS STUDY ……………….. 1<br>FAMILY MEMBER ………... 2<br>FRIEND …………………….. 3<br>HEALTH WORKER ……...... 4<br>OTHER (specify:________) .. 5	
3	Has a health worker ever discussed hypertension or its treatment with you?	YES ………………….……… 1<br>NO ………………………..… 2	
4	What do you think hypertension is?	HIGH BP …………………..… 1<br>EXCESSIVE STRESS/WORRY .. 2<br>LACK OF ATTENTION …..… 3<br>OTHER (specify:________) ... 4<br>DON'T KNOW …………..….. 5	
5	Is hypertension hereditary?	YES ………………….……… 1<br>NO ………………………..… 2<br>DON'T KNOW …………….. 3	

6	Can hypertension be caused by food poisoning?	YES …………………….……… 1<br>NO ………………………..… 2<br>DON'T KNOW …………….. 3	
7	Can hypertension be caused by bad spirits?	YES …………………….……… 1<br>NO ………………………..… 2<br>DON'T KNOW …………….. 3	
8	Can eating meat increase the risk for hypertension?	YES …………………….……… 1<br>NO ………………………..… 2<br>DON'T KNOW …………….. 3	
9	Can smoking increase the risk for hypertension?	YES …………………….……… 1<br>NO ………………………..… 2<br>DON'T KNOW …………….. 3	
10	Can drinking alcohol increase the risk for hypertension?	YES …………………….……… 1<br>NO ………………………..… 2<br>DON'T KNOW …………….. 3	
11	Can exercising increase the risk for hypertension?	YES …………………….……… 1<br>NO ………………………..… 2<br>DON'T KNOW …………….. 3	

12	What are the symptoms of hypertension? (select all that apply)	HEADACHE ……………..…. 1<br>PALPITATIONS ………..…..... 2<br>FEVER …………………...…. 3<br>CHEST PAIN …....………….... 4<br>FEELING FAINT ………...….. 5<br>SUDDEN COLLAPSE ……..... 6<br>SUDDEN STROKE ……….... 7<br>NO SYMPTOMS ...……….…. 8<br>OTHER (specify:________) .. 9<br>DON'T KNOW …………..... 10	
13	Can one have hypertension without displaying any signs or symptoms?	YES …………………..……… 1<br>NO ………………………..…. 2	
14	Can hypertension be controlled with treatment?	YES …………………..……… 1<br>NO ………………………..…. 2	→ 19
15	Which of the following treatments should be used to treat hypertension?	TRADITIONAL MEDICINE .. 1<br>ORTHODOX MEDICATION.. 2<br>HERBAL REMEDIES...…...... 3<br>OTHER (specify:________) ... 4	
16	Does treatment involve changing your lifestyle?	YES …………………..……… 1<br>NO ………………………..…. 2	
17	When treated, is hypertension cured once and for all?	YES …………………..……… 1<br>NO ………………………..…. 2	

18	How long is treatment required for hypertension?	A FEW DAYS ……………..... 1<br>1-3 WEEKS …....……….….... 2<br>1 MONTH ………………....... 3<br>2-11 MONTHS ……………….. 4<br>1 YEAR ……………..…..... 5<br>2+ YEARS ……………….….. 6<br>INDEFINITELY ……………..... 7<br>DON'T KNOW ……………….. 8	
19	Are there consequences of untreated hypertension?	YES ……………….………. 1<br>NO ……………………...…. 2	→ 21
20	What are the consequences of untreated hypertension?	STROKE ……………….….... 1<br>HEART FAILURE ………..… 2<br>KIDNEY FAILURE ………… 3<br>DEATH ……………………… 4<br>OTHER (specify:________) .. 5<br>DON'T KNOW ……………...... 6	
21	Do you have hypertension?	YES …………………………..…1<br>NO ……………………...…. 2<br>DON'T KNOW ……………....3	→ 26<br>→ 26

Section 2: Attitudes and Lifestyle Practices Regarding Hypertension

NO.	QUESTIONS AND FILTERS	CODING CATEGORIES	SKIP?
22	Do you take medication for hypertension?	YES (specify:___)...…..……… 1<br>NO ………………………..… 2	 → 25
23	How long have you been on hypertension medications?	< 1 MONTH ……..……….…... 1<br>2-6 MONTHS ………..…..… 2<br>1+ YEAR …………………..... 3<br>DON'T REMEMBER ……..... 4	
24	Have you taken the medication regularly over the last 2 weeks?	YES ……………………..……… 1<br>NO ………………………..… 2	
25	Do you use any other treatments for your hypertension?	LIFESTYLE CHANGE …….. 1<br>HERBAL REMEDIES……..... 2<br>TRADITIONAL MEDICINE . 3<br>OTHER (specify:________) .. 4	
26	Are you concerned with your overall health?	YES ……………………..……… 1<br>NO ………………………..… 2<br>SOMEWHAT …………..…..... 3	
27	Are you on any (other) medications?	YES (specify: ____)…........… 1<br>NO ………………………..… 2	
28	Do you attend clinics regularly?	YES ……………………..……… 1<br>NO ………………………..… 2	 → 31
29	How often do you attend clinics?	2-4 TIMES A MONTH …...…. 1<br>MONTHLY ……………….…... 2<br>6-11 TIMES A YEAR …...….. 3<br>2-6 TIMES A YEAR ………... 4<br>YEARLY …………………..... 5	
30	Why do you typically attend the clinic?	ROUTINE VISIT …………..... 1<br>SICK/INJURY VISIT ….…..... 2<br>MATERNAL + CHILD CARE ….. 3	

		OTHER (specify: _____) ….... 4	
31	Do you use alternative (traditional) medicines?	YES …………………….……… 1<br>NO ………………………..… 2	
32	Have you had your blood pressure checked?	YES …………………….……… 1<br>NO ………………………..… 2	→ 36
33	When was the last time you had your blood pressure checked?	TODAY ………………….….. 1<br>< 1 MONTH AGO ………..... 2<br>2-6 MONTHS AGO ……..… 3<br>1+ YEAR AGO ……………... 4<br>DON'T REMEMBER …..…... 5	
34	How often do you get your blood pressure checked?	2-4 TIMES A MONTH ……... 1<br>MONTHLY ……………….... 2<br>6-11 TIMES A YEAR …….... 3<br>2-6 TIMES A YEAR …..…... 4<br>YEARLY ………………….... 5<br>OTHER (specify: _____) …... 6	
35	Is your blood pressure well controlled?	YES …………………….……… 1<br>NO ………………………..… 2<br>DON'T KNOW ………………3	
36	Do you take salt with your food?	ALWAYS ……..…………… 1<br>SOMETIMES ……..…….... 2<br>NEVER ……………………...... 3	
37	Do you cook your food with oil?	ALWAYS ……..…………… 1<br>SOMETIMES ……..…….... 2<br>NEVER ……………………...... 3	

38	How often do you eat fruits and vegetables?	6+ TIMES A DAY ………..… 1<br>5 TIMES A DAY….. ……..… 2<br>1-4 TIMES A DAY ……….... 3<br>1-5 TIMES A WEEK ……..... 4<br>NEVER ……………………..… 5	
39	How often do you eat meat?	EVERY DAY ……………..… 1<br>2-5 TIMES A WEEK ……..… 2<br>ONCE A WEEK …………..... 3<br>NEVER …………………….... 4	
40	How often do you eat rice?	EVERY DAY ……………….. 1<br>2-5 TIMES A WEEK ……..…. 2<br>ONCE A WEEK ……………. 3<br>NEVER …………………….... 4	
41	Do you drink alcohol?	YES ………………….……… 1<br>NO ………………………..… 2	→ 43
42	How often do you drink alcohol?	EVERY DAY ……………..…. 1<br>2-5 TIMES A WEEK ……..…. 2<br>ONCE A WEEK …………..… 3	
43	Do you smoke?	YES ………………….……… 1<br>NO ………………………..… 2	→ 45
44	How often do you smoke?	EVERY DAY ……………..…. 1<br>2-5 TIMES A WEEK ……….. 2<br>ONCE A WEEK …………..… 3	
45	Do you exercise regularly?	YES ………………….……… 1<br>NO ………………………..… 2	→ 48
46	How often do you exercise?	3+ HOURS/WEEK ..…...……. 1<br>1-2.9 HOURS/WEEK ……..…. 2<br>0-1 HOURS/WEEK ……….… 3<br>NEVER …………………….... 4	

47	What do you do for exercise?	WALK ……………………..…. 1<br>RUN ………………….….….. 2<br>SPORTS ……………………..…. 3<br>OTHER (specify:________) ... 4	
48	Do you try to control your weight?	YES ………………….……… 1<br>NO ……………………..… 2	
49	How often do you think about your health?	ALWAYS ……..……………… 1<br>SOMETIMES ……..……….. 2<br>NEVER ………...…………..... 3	
50	How often do you think about the health of your family?	ALWAYS ……..……..…....… 1<br>SOMETIMES ……….…..….. 2<br>NEVER ……………...……..... 3	

Appendix 2: Focus Group Field Guide

The following field guide outlines the various topics and guiding questions that will be used to run the focus group discussions about hypertension.

Perceptions

1. How do you perceive your own health? Your family's?
2. What is hypertension?
3. How do you feel about needing to control your blood pressure?
4. What resources do you have to take care of your health and blood pressure?
5. Do you think you are capable of controlling and managing your blood pressure?
6. Do you think your community is healthy?

Taboos

1. Are you concerned with what others will think about your health?

2. How do you feel about seeking out health care?
3. Where would you go to seek out health care?

Misconceptions

1. What causes hypertension?
2. Do you think you can prevent hypertension? How?
3. Do you think you can cure hypertension? How?

Questions

1. What kinds of questions do you have about your health?
2. Do you feel like you have the knowledge to manage your own health?
3. What other information do you need?
4. Do you want to learn more about hypertension?
5. How would you like to receive information about your health?
6. What support/resources do you need to practice healthier lifestyles?

Concerns

1. What are your biggest health concerns?
2. What are your biggest concerns for your family's health?
3. Do you think hypertension is concerning?
